# Investigating the effect on biogenic amines, nitrite, and N-nitrosamine degradation in cultured sausage ripening through inoculation of *Staphylococcus xylosus* and *lactic acid bacteria*

**DOI:** 10.3389/fmicb.2023.1156413

**Published:** 2023-03-09

**Authors:** Panpan Hu, Urooj Ali, Tariq Aziz, Li Wang, Jianying Zhao, Ghulam Nabi, Manal Y. Sameeh, Yanqin Yu, Yingchun Zhu

**Affiliations:** ^1^Department of Life Science, Lyuliang University, Lishi, Shanxi, China; ^2^Department of Biotechnology, Quaid e Azam University, Islamabad, Pakistan; ^3^School of Food and Biological Engineering, Jiangsu University, Zhenjiang, China; ^4^Institute of Nature Conservation, Polish Academy of Sciences, Krakow, Poland; ^5^Department of Chemistry, Faculty of Applied Sciences, Al-Leith University College, Umm Al-Qura University, Makkah, Saudi Arabia; ^6^College of Food Science and Engineering, Shanxi Agricultural University, Taigu, Shanxi, China

**Keywords:** starter cultures, fermented sausages, nitrite degradation, biogenic amines degradation, N-nitrosamine degradation

## Abstract

**Introduction:**

Microbial inoculants can reinvent the value and edible security of cultured sausages. Various studies have demonstrated that starter cultures made up of *Lactic acid bacteria* (LAB) and *Staphylococcus xylosus* (known as L-S) isolated from traditional fermented foods were used in fermented sausage manufacturing.

**Methods:**

This study evaluated the impact of the mixed inoculation cultures on limiting biogenic amines, nitrite depletion, N-nitrosamine reduction, and quality metrics. Inoculation of sausages with the commercial starter culture (SBM-52) was evaluated for comparison.

**Results and discussion:**

Results showed that the L-S strains could rapidly decrease the water activity (Aw) and pH of fermented sausages. The ability of the L-S strains to delay lipid oxidation was equivalent to the SBM-52 strains. The non-protein nitrogen (NPN) contents of L-S-inoculated sausages (0.31%) were higher than that of SBM-52-inoculated sausages (0.28%). After the ripening process, the nitrite residues in the L-S sausages were 1.47 mg/kg lower than in the SBM-52 sausages. Compared to the SBM-52 sausages, there was a 4.88 mg/kg reduction in the biogenic amines’ concentrations in L-S sausage, especially for histamine and phenylethylamine concentrations. The N-nitrosamine accumulations of the L-S sausages (3.40 ug/kg) were lower than that of the SBM-52 sausages (3.70 ug/kg), and the NDPhA accumulations of the L-S sausages were 0.64 ug/kg lower than that of the SBM-52 sausages. Due to their significant contributions to nitrite depletion, biogenic amine reduction, and N-nitrosamine depletion in fermented sausages, the L-S strains have the potential to serve as an initial inoculant in the process of manufacturing fermented sausages.

## 1. Introduction

For centuries, fermented food has been the main component of the human diet (Aziz et al., 2022). *Lactic acid bacteria* (LAB) is a crucial and predominantly used bacteria in the food sector, and has been generally regarded as safe (GRAS; Sarwar et al., 2018; Aziz et al., 2022, 2023). LAB strains have been widely studied for a long time for their widespread beneficial properties, which are useful for living organisms (Jian et al., 2017, 2020; Zhang et al., 2020, 2022; Aziz et al., 2020a,b,c, 2021). The most noteworthy cultured meat items in China, are fish, sausages, and ham (Wang et al., 2022). Fermented sausages are somewhat cultural meat with a rich tradition in China and are preferred by consumers for their idiosyncratic flavors and rich nutrition (Luan et al., 2021). However, with the increasing consumers’ attention to the safety of fermented sausages, how to reduce the concentration of some harmful substances in the fermented sausages, such as biogenic amines (BAs), residual nitrite and N-nitrosamine, has motivated researchers’ interest.

Technologically, fermented sausages are produced by fermenting for a couple of days, succeeded by a lengthy ripening stage that can last up to months to promote the production of various properties (Chen et al., 2016). However, the high occurrence of biogenic amines (BAs) and residual nitrite has been discovered in fermented sausages due to additives and microbial contamination (Wang et al., 2016). Nitrite has long been used as a necessary curing agent. In fermented sausages, it plays a variety of roles, such as antibacterial effects, color fixation, texture improvement, and effective antioxidants (Wang et al., 2013). However, Nitrite is a critical precursor to N-nitrosamines (Oh et al., 2004), which could react with amines to produce carcinogenic, mutagenic, and teratogenic nitrosamines (Wang et al., 2016; Kononiuk and Karwowska, 2020). BAs are generated by amino acids (AAs) decarboxylation due to microorganisms with certain amino acid decarboxylase activities (Hao and Sun, 2020). The major BAs found in fermented sausages are tyramine, histamine, and tryptamine, which could trigger adverse health effects, including headaches, allergic reactions, and high blood pressure (Schirone et al., 2022). Therefore, reducing nitrites and BAs contents is of applied importance for reinventing the quality and edible security of fermented sausages.

Recently, the application of starter cultures has received attention as a potential means of guaranteeing the high value and edible security of fermented sausages (Lorenzo et al., 2014; Xiao et al., 2020). In fermented sausages, the main bacterial inoculation cultures consist of cocci that are Gram-and catalase-positive (GCC+), from the *Staphylococcaceae* and *lactic acid bacteria* (LAB) (dos Santos Cruxen et al., 2019). Literature showed that LAB and *Staphylococcus xylosus* possess nitrite reductases that could reduce nitrite contents (Chen et al., 2016; Wang et al., 2016). LAB also produces lactic acid that decreases the pH, and the nitrite degradation rate increases with the appropriate decrease in pH (Liao et al., 2019). Sun et al. (2016) found that nitrite residues in fermented fish products inoculated with *S. xylosus* 135 and *L. plantarum* 120 decreased by 6 and 70 μg/kg, respectively, compared with the blank group. A similar analysis was previously described by Chen et al. (2016), who discovered that the nitrite concentration of *L. sakei* CMRC15 or *L. plantarum* CMRC6-inoculated sausages was considerably lower while ripening than non-inoculated sausages. Inoculation with starter bacterial cultures harboring bacteria with oxidase activity of the BAs but no decarboxylase activity of the AAs is one successful way to limit BAs buildup in fermented sausages (Sun et al., 2016; Ebrahimi and Farshidi, 2019), so the selection of strains is crucial to the BAs degradation. It has been reported that inoculation of both *L. plantarum* and *S. xylosus* as inoculants aided in decreasing BAs in cultured sausages (Sun et al., 2016). Nie et al. (2014) argued that a combined inoculation culture containing *Saccharomyces cerevisiae* and *Lactobacillus plantarum* that could reduce cadaverine and putrescine concentrations in fermented silver carp sausages. In general, N-nitrosamine formation is attributed to the interaction of a nitrosation agent such as sodium nitrite, and a secondary amine (Flores et al., 2019). The existence of BAs and other substances for degrading proteins is regarded as a significant source of amine precursors. Adding a microbial is considered a potential solution for decreasing the precursors (amines and nitrites) to deplete N-nitrosamine formation. Sun et al. (2023) found that *L. plantarum*X22-2 and HX2-1, *Pediococcus pentosaceus* X6-6 could effectively degrade seven kinds of BAs, especially tryptamine and phenethylamine. Therefore, a mixture of *Staph.* and LAB species were beneficial in improving that fermented sausages are safe to eat.

Based on our previous studies, the nitrite-degrading and BAs-degrading strains (including *Pediococcus pentosaceus* M GC-2 andM SZ1-2, *Staphylococcus xylosus* Y CC-3 and *Lactobacillus plantarum* M SZ2-2) were selected from traditional Chinese fermented foods through morphological identification, genome sequencing, and physicochemical experiments (Li et al., 2020). The mixed starter cultures [(M)-SZ1-2, SZ2-2, -GC-2, and Y CC-3] at a ratio of 1:1:1:6 known as *Lactobacillus-Staphylococcus* or L-S and the commercial starter culture SBM-52 were inoculated in fermented sausages, respectively. Quality parameters of fermented sausages, mainly Thiobarbituric Acid Reactive Substances (TBARs), pH, color water activity (Aw), total volatile base nitrogen (TVB-N), and non-protein nitrogen (NPN) were observed. The impact of different inoculant cultures on nitrite depletion, BAs degradation, and N-nitrosamine reduction of fermented sausages was also tested.

## 2. Methodology

### 2.1. Materials

The chemicals trichloroacetic, thiobarbituric acid, methylene chloride, and chloroform were obtained from Tianjin Tianli Chemical Reagent Co., Ltd., a company based in Tianjin, China.

### 2.2. Strains and starter culture

The bacteria *S. xylosus* Y CC-3, M GC-2 and M SZ1-2 from P. pentosaceus, and M SZ2-2 from *L. plantarum*, were retrieved from local Chinese fermented products such as bacon products, sausages, and sour porridges. They were discovered using 16S rDNA sequencing technology and preserved at Shanxi Agricultural University’s Meat Research Center in China. The microbes were then cultivated for 3 days at 37°C in broth culture supplied by Qingdao Haibo Biotechnology Company Limited. The bacterial strains were collected after a 10-min centrifugation at 8,000 g using an Eppendorf Technology Company’s 5804R equipment, then treated with 0.85% saltwater twice and kept at 4°C for subsequent use.

### 2.3. Preparation of fermented sausages

Fresh Back fat (30%) and lean pork (70%) were purchased at the JiaJiaLi marketplace in Taigu, China, to make fermented sausages. The pork was minced (MM-12, Shaoguan Xintongli Foodstuff Machinery Co., Ltd., Shaoguan, China) and preserved (via pickling) for a day at 4°C with a combination of sodium tripolyphosphate (0.3%), salt (2.8%), sodium nitrite (0.015%), and ascorbate (0.05%). The seasoned meat was then mixed with sugar (5%), chopped fat, monosodium glutamate (0.6%), pepper (0.1%), and five-spice powder (0.6%). The ground meat was split into two identical batches: Batch A was treated with the commercially used SBM-52 starting culture (*S. xylosus*, *P. pentosaceus*, *S. carnosus*, and *P. acidilactici*) (from Danisco Technologies Corporation, Italy), with an additional quantity of SBM-52 inoculant at 10^7^ cfu/g, according to the SBM-52 manual. Batch B received a mixed L-S starting culture (*P. pentosaceus* M GC-2, M SZ1-2, *S. xylosus* Y CC-3, and *L. plantarum* M SZ2-2), in a 1:1:6:1 ratio, with an additional 10^7^ cfu/g (M GC-2: M SZ1-2: Y CC-3: M SZ2-2) that is (1.11:1.11: 6.66: 1.11) × 10^6^ cfu/g. The ingredients were packed into natural pig cases with a thickness of about 2 cm (available from Nantong Jiangsu’s Hengyu Casing Direct Distributor, China), and the sausages were put in an incubation container (model: LRHS-150, from Shanghai’s Yuejin Medical Equipment Factory, China). These were then fermented at 30°C with a relative humidity of 90% till pH 5.1 and then matured for 2 weeks at 15°C with a moisture content of 65%. The cultured sausages were sampled randomly after zero (when the fermentation finished), 1, and every 3rd day of maturation (4, 7, 10, 13) for physicochemical qualities and buildup of nitrite, BAs, and N-nitrosamines.

### 2.4. Determination of pH

The sausages’ pH was determined with a digital pH meter (ST2100, Ohouse Inc., New Jersey, United States) and the method described by GB5009.237–2016 (China).

### 2.5. Determination of Aw

Wuxi Huake Instrument Company, based in Jiangsu, China, supplied a water activity monitor used to calculate Aw.

### 2.6. Determination of color

A colorimeter (CM-5, Konica Minolta, Japan) was used to measure the a* (redness), L* (lightness), and b* (yellow-ness) indices of the cultured sausages. Before the analysis, the instrument was standardized with a white plate (a* = 0.50, L* = 92.00, b* = 11.28), and the measurements for a*, L*, and b* were obtained for every specimen.

### 2.7. Determination of TBARs

The concentrations of TBARS in cultured sausages were determined using a technique published previously by Li et al. (2020). Each sausage sample (5 g) was blended and purified in trichloroacetic acid (15 mL, 7.5% w/v). 2.5 mL (0.02 M) thiobarbituric acid was blended with 2.5 mL of filtrate in test tubes. After being introduced to 3 mL of chloroform, the mixture was put in a bath of boiling water for 40 min, cooled down to room temperature, then spun at 2°C for 10 min (2,000 g). The supernatant was extracted, and its absorption was determined at 532 nm. The TBARS levels were reported in mg/kg (or mg MDA/kg).

### 2.8. Assessing the TVB-N content

The TVB-N content was assessed using the semi-microscopic nitrogen determination technique outlined in the Determination of Volatile Salt-based Nitrogen in Foods (GB 5009.228-2016, China). The assessment outcome was reported in terms of mg/100 g.

### 2.9. Determination of NPN

N-nitrosamines (NPN) contents in cultured sausages were measured using a technique established by Lobo et al. (2016). The sample (10 g) was pulverized twice in deionized water (50 mL) before being centrifuged for 10 min at 5,000 g, and 4°C. The supernatant was then combined with 20% trichloroacetic acid (25 mL) at room temperature for 30 min before being centrifuged. The solution was purified *via* Whatman No. 4 filter paper, and the NPN content was calculated using the Kjeldahl technique (AOAC, 2000).

### 2.10. Nitrite assessment

The Nitrite level present in the fermented sausages was evaluated by following the guidelines of GB5009.33 (2016) method.

### 2.11. Assessment of BAs

The BAs content in the sausages was determined by adhering to the protocol outlined in GB5009.237-2016 (China). The outcome of the test was reported in mg/100 g.

### 2.12. N-nitrosamines’ assessment

A modified version of the method developed by Sun et al. (2017) was used to detect N-nitrosamines’ presence in the samples. To prepare standard solutions, Nitrosamines were dissolved in methylene chloride (200 μg/mL), and working solutions at concentrations of 0.125 multiples (0 × 0.0125, 1 × 0.0125, ……, 5 × 0.0125) μg/mL were created in methylene chloride and stored at 4°C for later use. To extract the nitrosamines, 200 g of cut sausage samples were combined with 100 mL of distilled water and 120 g of NaCl in a distilling flask. The receiving flask was filled with 40 mL of dichloromethane and a small amount of ice. The 400 mL of collected distillate was combined with 80 g of NaCl and 3 mL of H2SO4, shaken for 5 min, and then put into a 500 mL separating funnel and extracted three times with 180 mL dichloromethane of chromatographic grade. The samples were dried with sodium sulphate (anhydrous), concentrated, using the Kuderna-Danish apparatus, to 3–5 mL, and then vaporized under nitrogen to a total volume of 1 mL. N-nitrosamines were assessed using high-performance liquid chromatography (HPLC). Sample (10 mL) was introduced to a 1260ALS HPLC, from Agilent Ltd., United States, equipped with a C18 column (5 μm; 4.6 × 250 mm) and adjusted to 25°C and 1.0 mL/min flow rate. Eluent A or acetonitrile was used in the mobile phase.

### 2.13. Statistical analysis

The research findings were reported as the mean value standard error computed after the study was repeated thrice. Charts made using OriginPro 9.0 program were used to illustrate the data (Origin Lab, Northampton, MA, United States). Statistix 8.1 (Tallahassee, United States) was used to evaluate statistical significance that was set at *p* < 0.05.

## 3. Results and discussion

### 3.1. pH

**Figure 1:**
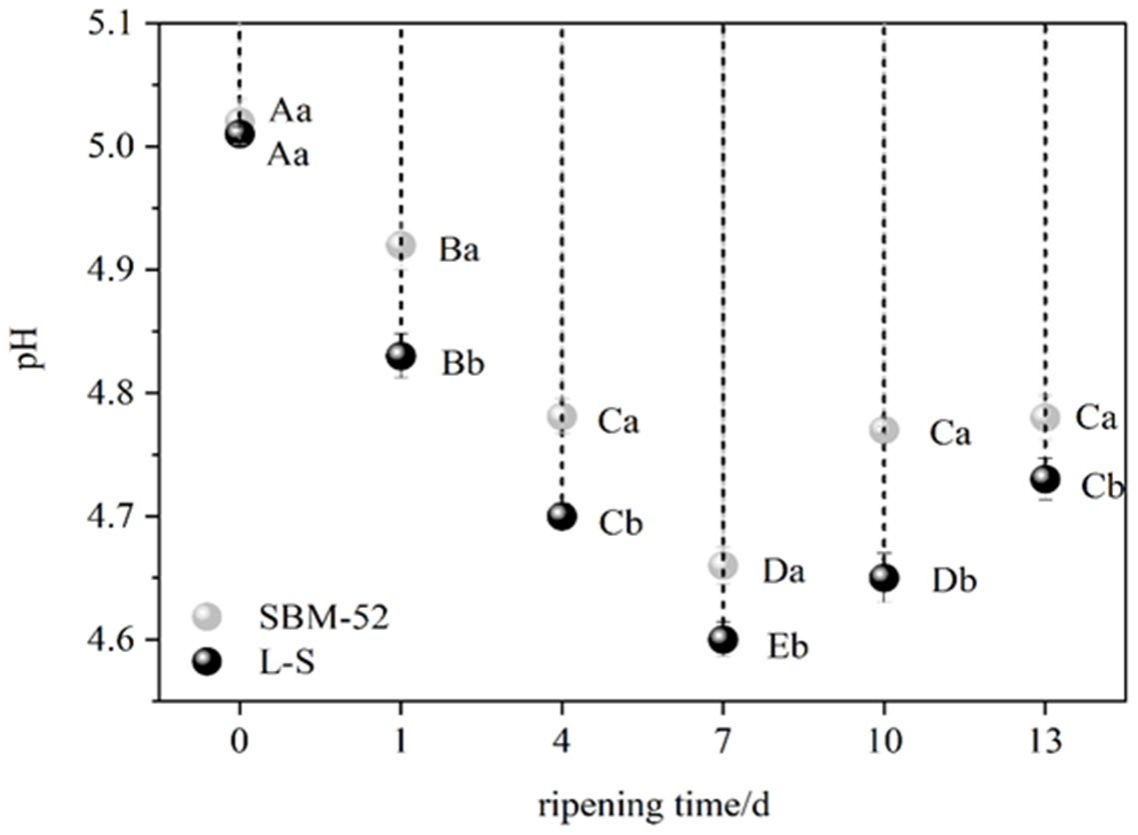
The impact of inoculating L-S and SBM-52 strains upon the pH of cultured sausages throughout the ripening phase. Different capital letters (A–E) at various times of the same sausages show significant changes (*p* < 0.05), whereas various lowercase characters (a–b) show considerable variation (*p* < 0.05) in different sausages.

The synthesis of enough organic acids during fermentation enables the sausages to achieve a pH below 5.1 (Agüero et al., 2020), then the sausages enter the ripening process. Variations in pH during the ripening process of the fermented sausages are represented in Figure 1. The starting pH was about 5.00, decreasing to 4.60 and 4.66 by day 7 of ripening in the L-S-inoculated sausages and the SBM-52-inoculated sausages, respectively. The bacterial activity-induced buildup of organic acids, especially lactic acid of LAB, might lower pH (Lorenzo et al., 2014; Ge et al., 2019). In particular, the pH values of the L-S group (4.60) were considerably reduced (*p* < 0.05) compared to the SBM-52 group (4.66) on day 7, confirming the L-S strain’s strong acidifying ability (Yu et al., 2021). Seven days after ripening, the pH levels in the two groups slowly climbed till the completion of the ripening, which was likely associated with the microbial-associated proteolytic activity. Bacterial proteases cause proteolytic degradation, leading to amines, amino acids, and peptides’ production for organic acid buffering (Ruiz-Moyano et al., 2011). Throughout the ripening process, the pH of the L-S-cultured sausages was always reduced compared to the SBM-52-inoculated sausages; the results suggested that the L-S group can extend the fermented sausages’ shelf-life and ensure their safety because the lower pH values potentially stifle the expansion of adverse microorganisms (especially spoilage microbiota and pathogens) and prevent the accumulation of biogenic amines (Neffe-Skocińska et al., 2020).

### 3.2. Aw

**Figure 2:**
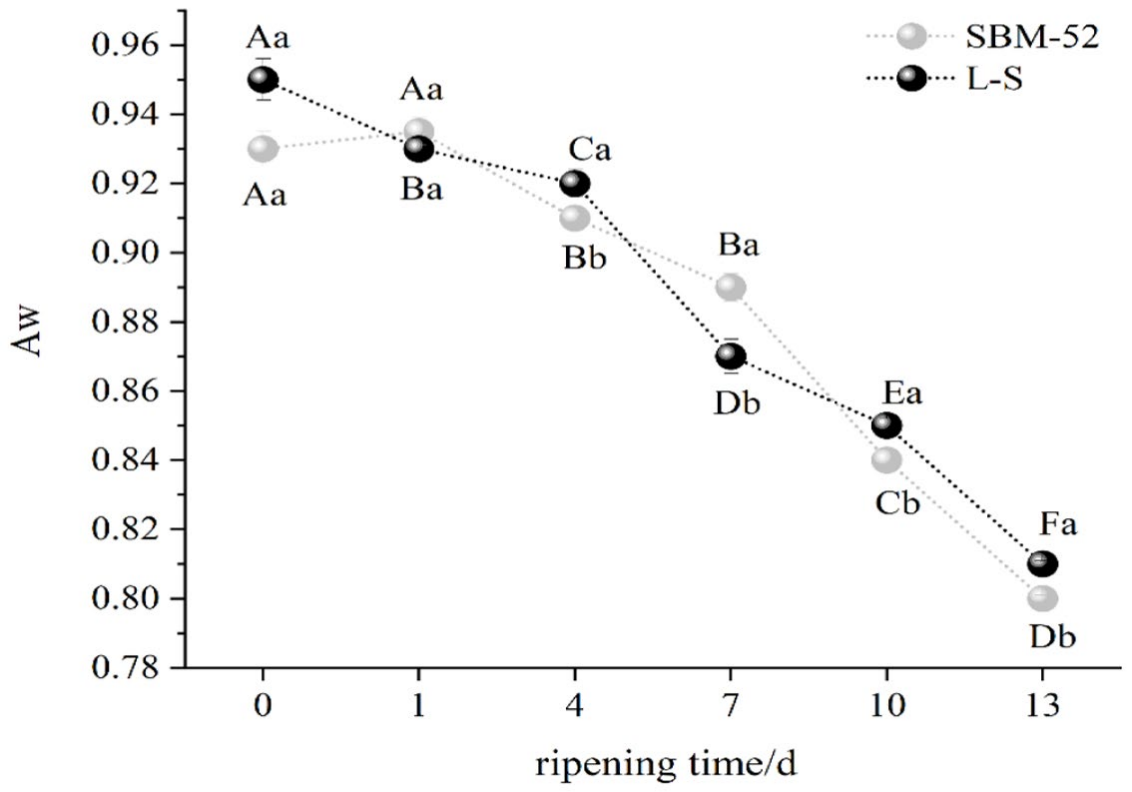
The influence of culturing L-S and SBM-52 strains on the Aw levels of cultured sausages throughout the ripening phase was investigated.

Aw (water activity) affects the shelf-life of products entirely, and a low Aw helps to improve the storage stability of foods (Seleshe et al., 2021). Variations in the Aw values in all sausages were given in Figure 2, the Aw values of the L-S-inoculated sausages and the SBM-52-inoculated sausages decreased sharply (*p* < 0.05) by 15 and 14%, respectively, throughout the ripening process. Our findings are consistent with those published by Xiao et al. (2020), who also experimented on the culturing of *Lactobacillus plantarum* and *Staphylococcus xylose* in Chinese dry-fermented sausage lowered their Aw values. Changes in Aw values were most likely related to pH change, and the decrease in pH while ripening caused the denaturation of certain muscle protein acids, and then reduced the water-holding capacity of the protein (Sun et al., 2016). Furthermore, the environment’s relative humidity being reduced also resulted in the reduction of the Aw of the fermented sausages (dos Santos Cruxen et al., 2018). At 13 days, the final Aw values of the L-S sausages and the SBM-52 sausages were 0.81 and 0.80, respectively. The Aw in all sausages decreased to below 0.81 on day 13th, and most microorganisms might cease to grow (Xiao et al., 2020), so the sausages were considered acceptable for food. A lower Aw is beneficial to sausages’ safety and shelf-life extension.

### 3.3. Color

**Figure 3:**
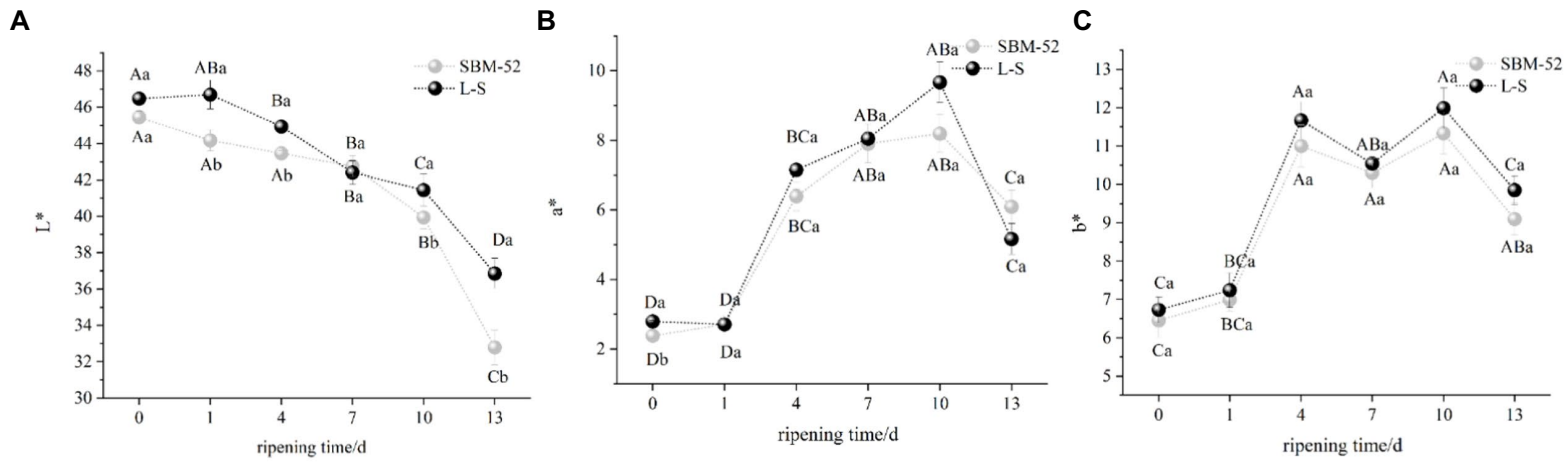
The influence of culturing L-S strains and SBM-52 strains on the L* **(A)**, a* **(B)**, and b* **(C)** values of fermented sausages throughout the ripening process.

To understand the optical properties of the fermented sausages, color parameters L*(lightness), a*(redness), and b*(yellowness) were investigated. As shown in Figure 3A, the L* values of the two groups exhibited a significant (*p* < 0.05) decrease throughout the ripening process, which might be linked to the fact that the meat protein closed to the isoelectric point with the decrease in pH, which made the surface of the sausages drier (Seleshe et al., 2021). It was noteworthy that the final values of L* of the L-S sausages were higher than that of the SBM-52, confirming that the L-S effectively contributed to the lightness of fermented sausages. For determining color, color stability, red color characterization, the a* parameter is the most sensitive (Garcia-Esteban et al., 2003). In Figure 3B, within the first 10 days of ripening, the a* values of the L-S sausages and SBM-52 sausages increased rapidly (*p* < 0.05) to 9.67 and 8.19, respectively. The rise in a* might be due to the metabolism of microorganisms. LAB and Staphylococcus participated in the degradation of nitrite to nitrogen monoxide, promoting the formation of nitrosomyoglobin, which caused the increase in redness (Chen et al., 2021). After 10 days, the a* values of the two groups gradually decreased, which could be attributed to the reduction of *Micrococcaceae* count. The catalase enzyme produced by *Micrococcacea* can degrade hydrogen peroxide (H_2_O_2_) generated by LAB. H_2_O_2_ attacks the heme pigments, which cause oxidative discoloration (Lorenzo et al., 2014; Ameer et al., 2021). As shown in Figure 3C, the b* values in both groups presented an increasing trend, compared to 0 days, the b* values in the SBM-52 sausages and the L-S sausages raised to 9.10 and 9.85, respectively, over the thirteen-day ripening. The increase in b* values could be explained by lipid oxidation (Lee et al., 2019; Liu et al., 2019). Although the a* value in L-S was lower than that in SBM-52 and the b* value was higher than that in SBM-52, there was no significant difference (*p* > 0.05) among the two groups at 13 days of ripening.

### 3.4. TBARs

**Figure 4:**
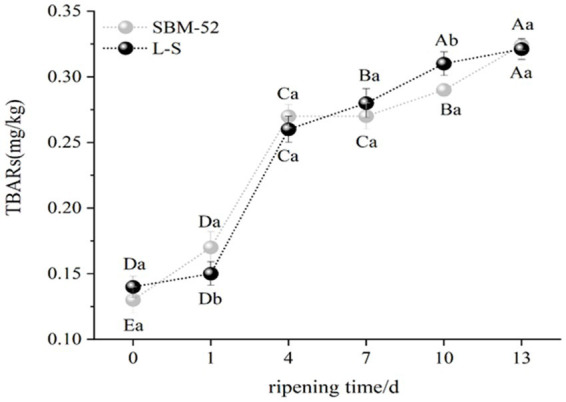
The impact of culturing L-S strains and SBM-52 strains on the TBARs levels of fermented sausages through ripening.

TBARs usually reflects the degree of lipid oxidation (Sun et al., 2017). An increase in TBARs as the ripening time in SBM-52 and L-S sausages was presented in Figure 4. However, there was no significant difference (*p* > 0.05) in TBARs values between the two groups at the beginning (0 day) and the end (13 days) of ripening, ranging from 0.16 mg/kg to 0.32 mg/kg in the L-S and 0.13 mg/kg to 0.32 mg/kg in the SBM-52. The results revealed that the anti-lipid oxidation ability of the L-S strains was consistent with that of the SBM-52. Our findings were in line with Chen et al. (2015), who reported that the inoculation of *P. pentosaceus* in fermented sausages played a key role in limiting lipid oxidation. The further explained by the fact that LAB and *Staphylococcus ssp.* have strong proteolytic activities, and certain active peptides could inhibit lipid oxidation (Wang et al., 2016; Zhu et al., 2019). The dates indicated that lipid oxidation in fermented sausages was effectively retarded by the L-S strains inoculation.

### 3.5. TVB-N

**Figure 5:**
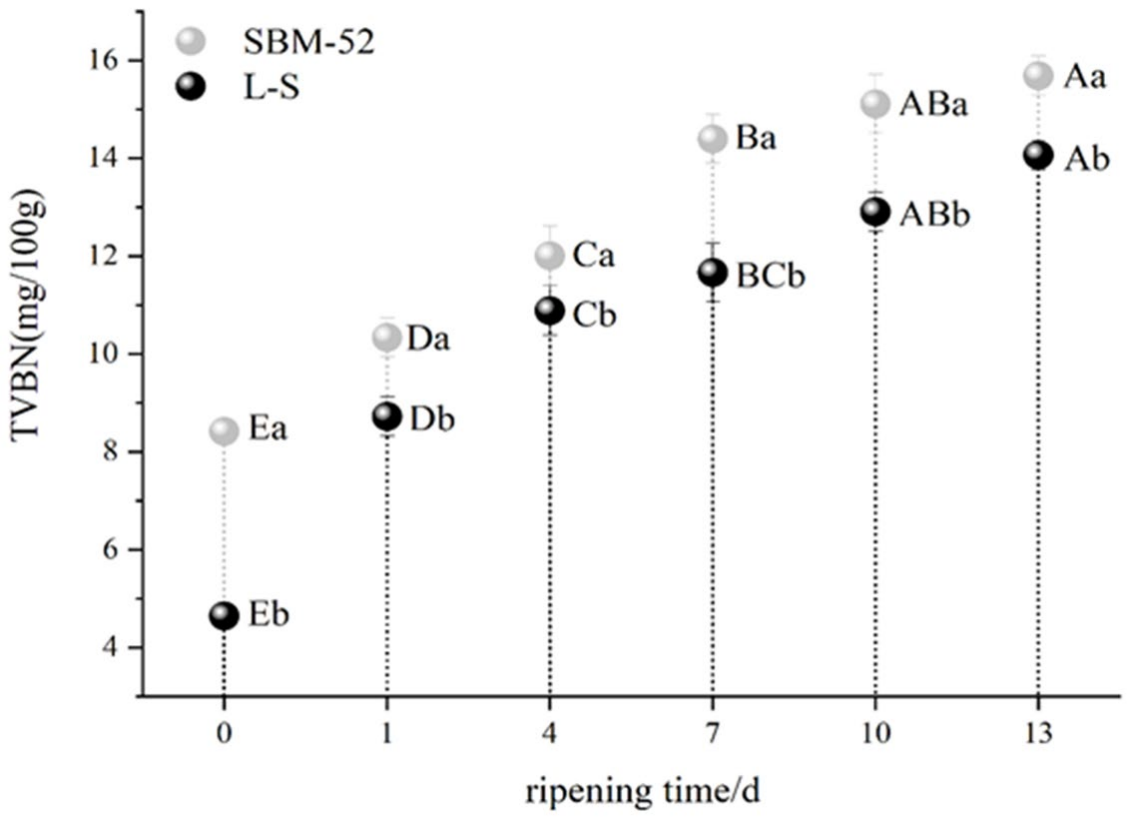
The influence of culturing L-S and SBM-52 strains on the TVB-N levels of fermented sausages through ripening.

The TVB-N composed of alkaline metabolites like ammonia and amines produced by the microbial decomposition of amino acids and proteins, is considered an important indicator for determining the protein degradation of meat products (Xiao et al., 2018; Wang et al., 2021). The changes in TVB-N values in both fermented sausages during the ripening period are presented in Figure 5. The fermented sausages in both groups showed an increased rapid (*p* < 0.05) trend during the ripening process. Lee et al. (2019) and Wang et al. (2021) reported that the increase in TVB-N values might be because of the combined actions of autolytic and microbiological deamination of amino acids and also by a complete microbial reduction of trimethylamine oxide to trimethylamine (Wang et al., 2021) suggested that *Enterobacteriaceae. app* is the primary producer of TVB-N. The TVB-N values of the L-S-inoculated sausages (14.07 mg/100 g) were obviously (*p* < 0.05) lower than that of the SBM-52-inoculated sausages (15.69 mg/100 g) at the end of the ripening process; the results showed that L-S strains help to inhibit the formation of *Enterobacteriaceae. app* in sausages.

### 3.6. NPN

**Figure 6:**
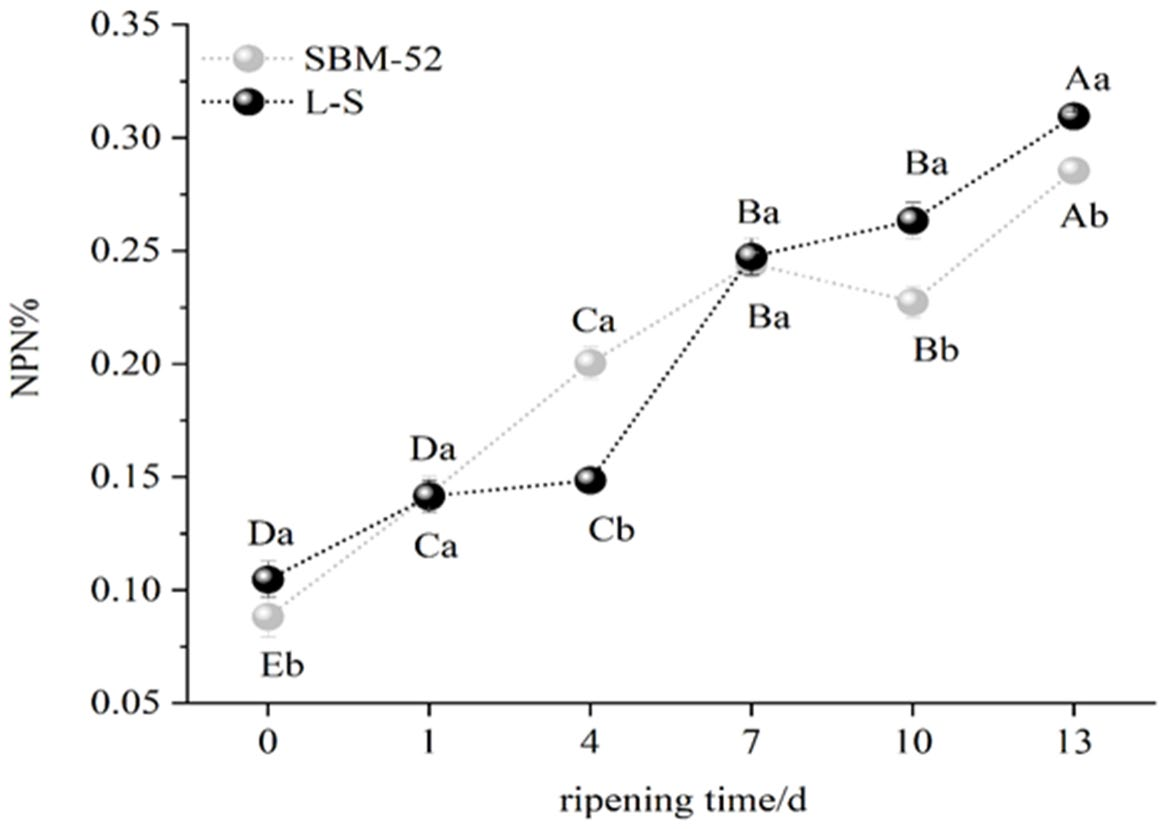
The impact of inoculating L-S strains and SBM-52 strains on cultured sausage’s NPN values.

NPN is made up of all of the nitrogen found in peptides, free amino acids, nucleic acids, and volatile basic nitrogen (Xu et al., 2018). As shown in Figure 6, the NPN values (% of total nitrogen) of the two groups showed an increasing trend, an increase in NPN is most likely related to the production of the endogenous proteases, in particular cathepsins B and D, which might promote the formation of the peptides and free amino acids (Yu et al., 2021). Other studies showed that proteolysis is characterized by an increase in NPN content during the processing of fermented sausages (Dalmış and Soyer, 2008). Furthermore, the NPN values of the L-S (0.31%) were obviously (*p* < 0.05) higher than that of the SBM-52 (0.28%) at the end ripening, which the different microorganism might further explain between the L-S group and the SBM-52 group. Our results are consistent with Fernández et al. (2016), who observed that using starter cultures (*Lactococcus* MS200 and *Staphylococcus* RS34) could increase NPN values.

### 3.7. Nitrite

**Figure 7:**
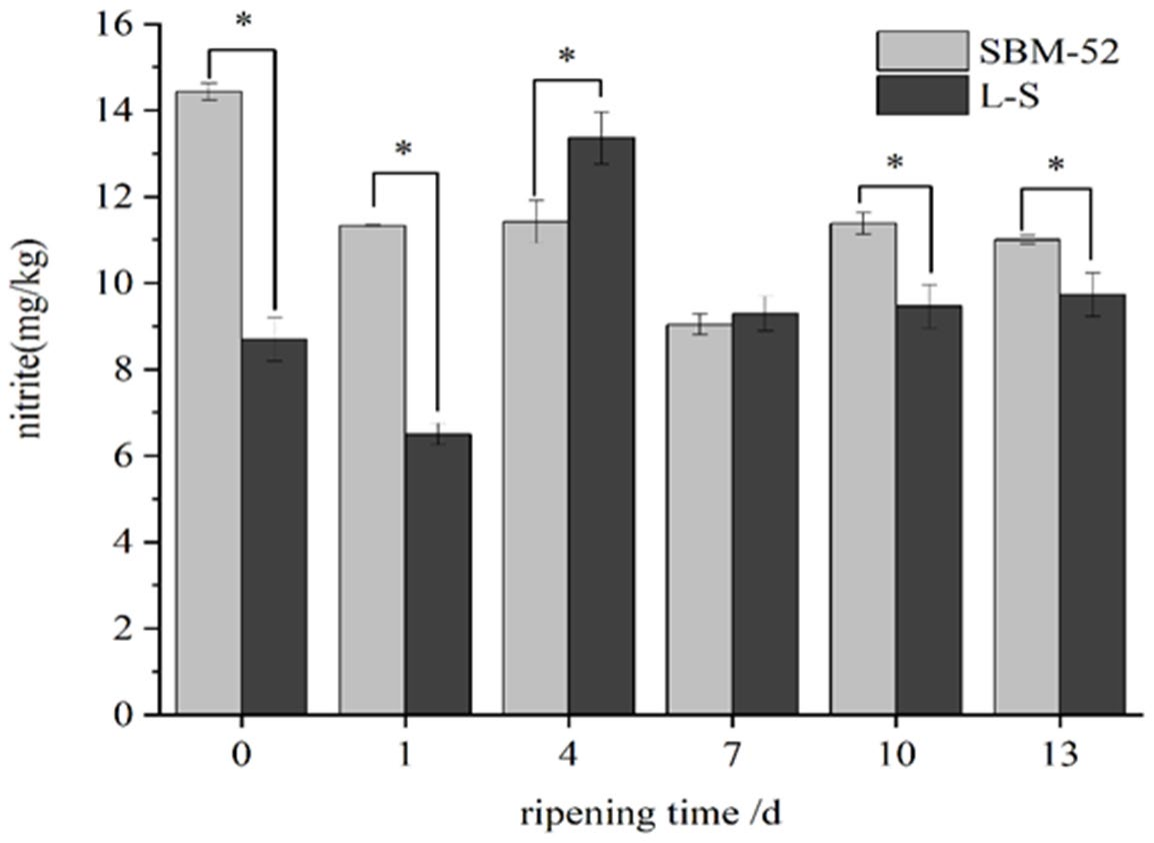
The influence of inoculating L-S and SBM-52 strains on remnant nitrite levels in cultured sausages throughout the ripening period. * Represents significant difference between groups.

The values of residual nitrite during the ripening process in fermented sausages were presented in Figure 7. In the first 7 days, the nitrite residues in the SBM-52 sausages dropped rapidly (*p* < 0.05) from 14.18 mg/kg (0 day) to 7.97 mg/kg (7 days), which might be explained by the fact that *Pediococcus pentosaceus* and *Lactobacillus acidilacticii* could produce the nitrite reductase (NiR) that involved in nitrite degradation (Paik and Lee, 2014). Chen et al. (2016) suggested that the formation of lactic acid led to a sharp pH decline and then contributed to nitrite reduction. After 7 days, the nitrite content increased slowly, likely associated with changes in pH. The contents of nitrite in the L-S sausages fluctuated during the whole ripening period. The nitrite contents decreased significantly (*p* < 0.05) at 0–1 days and 4–7 days. Based on previous experiments, the high activities of nitrite reductase were observed in M SZ1-2, M SZ2-2, and M GC-2 strains. Nitrite may be degraded to nitrogen oxides (NO, NO2, or N2O) by NiR (Xiao et al., 2018). A large amount of NiR produced by L-S strains during ripening could effectively reduce the nitrite contents of fermented sausages. A previous report also suggested that the *Lactobacillus plantarum* P2 isolated from fermented sauerkraut could degrade nitrite effectively (Chen et al., 2019). In addition, the nitrite and H^+^ undergo a reversible disproportionation reaction to produce nitrate (Hammes, 2012), reducing the nitrite contents of the fermented sausages. However, the nitrite content of sausages increased progressively at 1–4 days of the ripening process, which might be explained by the mutual transformation between nitrite and nitrate generated by disproportionation. At 7–13 days of ripening, the content of nitrite remained at 8 mg/kg. At the end of ripening, the content of nitrite in the L-S sausages (8.64 mg/kg) was significantly (*p* < 0.05) lower than that in the SBM-52 sausages (10.11 mg/kg). Our dates showed that the L-S starter culture could effectively reduce the nitrite contents of fermented sausages and ensure the edible safety of fermented sausages.

### 3.8. BAs

**Figure 8:**
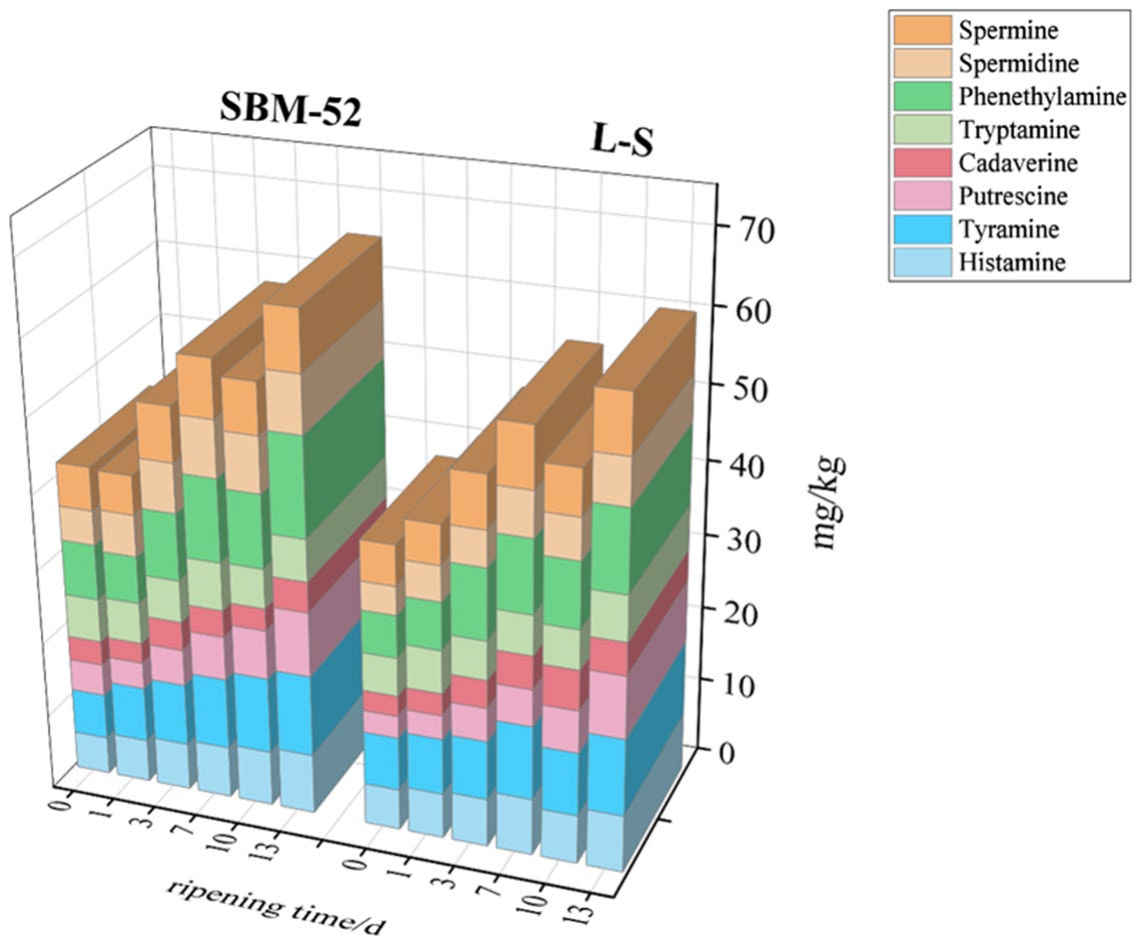
The impact of inoculating L-S strains and SBM-52 strains on residual nitrite levels in cultured sausages through the ripening process.

High contents of BAs in cultured sausages are produced by the decarboxylase activity of certain bacteria on free amino acids (FAA) (Schirone et al., 2022). Due to the toxicological effects, it constitutes a potential public health problem (Li et al., 2018). The assays of BAs concentrations (tyramine, cadaverine, histamine, putrescine, tryptamine, phenylethylamine, spermidine, and spermine) in both sausages were shown in Figure 8. The common biogenic amine intoxication was attributed to histamine and tyramine in fermented sausages (Zhu et al., 2020). Australia imposed limits on histamine content in food up to 200 mg/kg, South Africa and Europe (100 mg/kg), and FDA (50 mg/kg) (Azman et al., 2015; Li et al., 2018). The histamine concentrations in the two groups of fermented sausages were lower than the specified values. In the SBM-52 sausages, the histamine concentrations increased gradually from 5.00 mg/kg to 7.98 mg/kg during the maturation period. The concentrations of histamine in the L-S sausages were in the range of 5.43–7.60 mg/kg during the ripening process. It was noteworthy that the histamine concentrations of the L-S sausages (7.60 mg/kg) were considerably (*p* < 0.05) lower than the SBM-52 sausages (7.98 mg/kg) at the last stage of ripening. This might be attributed to the fact that the L-S strains screened by previous experiments did not contain the histidine decarboxylase (*hdc*) gene (Li et al., 2020) and then could suppress the formation of histamine. In addition, *Staphylococcus xylosus* possessed amine oxidase activity that helped to decrease histamine concentrations (Wang et al., 2016). Our results lined up with those given by Wang et al. (2016), who found that the histamine concentrations in cultured sausages inoculated with *S. xylosus, L. sakei,* and *P. pentosaceus* (0.32 mg/kg) were obviously reduced against the spontaneous fermentation sausages (8.85 mg/kg). Tyramine is the primary BAs observed in sausages during ripening. Initially, the tyramine concentrations in the SBM-52 sausages and the L-S sausages were 6.80 mg/kg and 7.13 mg/kg, rising to 10.83 mg/kg and 10.23 mg/kg, respectively, over the 13-day ripening. The growth rate of tyramine in the L-S sausages was lower than that in the SBM-52 sausages, indicating that the L-S strains strongly affected tyramine’s inhibition. A further explanation might be that the L-S strains did not have the tyrosine decarboxylase (*tdc*) gene. Zhu et al. (2020) also observed that the tyramine concentrations in sausages inoculated *Lactobacillus plantarum* was significantly lower than that in a blank group. The cadaverine and putrescine concentrations in the sausages cultured with L-S strains were 0.48 mg/kg and 1.28 mg/kg reduced, respectively, compared to the SBM-52 sausages at day 0 (*p* < 0.05). This might be explained by the presence of strains in the L-S sausages inhibiting putrescine and cadaverine synthesis throughout the fermentation. Sun et al. (2016) also discussed that *L. plantarum* and *S. xylosus* could lower the concentrations of cadaverine and putrescine. The two BAs concentrations in both groups showed fluctuating trends during ripening. There was no noteworthy difference (*p* > 0.05) in cadaverine and putrescine concentrations between the two groups on the 13th day of ripening, which indicated that the L-S strain had a similar inhibitory capacity against cadaverine and putrescine as SBM-52. Phenylethylamine is another primary BAs found in fermented sausages during ripening. The phenylethylamine concentrations rose rapidly during the 13 days, rising to 13.24 mg/kg and 10.81 mg/kg on day 13 for the SBM-52 and L-S sausages, respectively. The concentrations of tryptamine in both groups were maintained at less than 6 mg/kg during the whole ripening process. Spermidine and spermine are endogenous biological amines in meat products. Initially, spermidine and spermine concentrations in the L-S-inoculated sausages were 3.82 and 5.36 mg/kg, rising to 6.16 mg/kg and 7.74 mg/kg, respectively, over the 13-day ripening. Compared to the SBM-52 sausages, there was a 1.46 mg/kg and 0.32 mg/kg reduction in the spermidine and spermine concentration at day 13 (*p* < 0.05) in sausage inoculated with L-S strains. After 13 days of ripening, the total BA concentration in the L-S sausages (61.05 mg/kg) was apparently lower (*p* < 0.05) than that in SBM-52 sausages (65.93 mg/kg). The outcomes showed that the mixed strains could more successfully inhibit the accumulation of BAs. This might be attributed to the fact that the L-S strains contained producing amine-degrading enzymes (ADEs). Multicopper oxidase (MCO) and amine oxidase (AOs) were the most often observed ADEs in cultured items; however, unlike AOs, MCO may break down more than a single amine without creating hydrogen peroxide (Li and Lu, 2020). In earlier research, the genes producing MCO (D2EK17 and/or suf I) were found in L-S strains, but the genes producing decarboxylases (hdc and tdc) were not found (Li et al., 2020). The finding shows that the mixed L-S starting culture could successfully suppress BA formation. Sun et al. (2023) discovered that sausages infected with a mixed bacterial species may more effectively suppress BA formation.

### 3.9. N-nitrosamines

**Figure 9:**
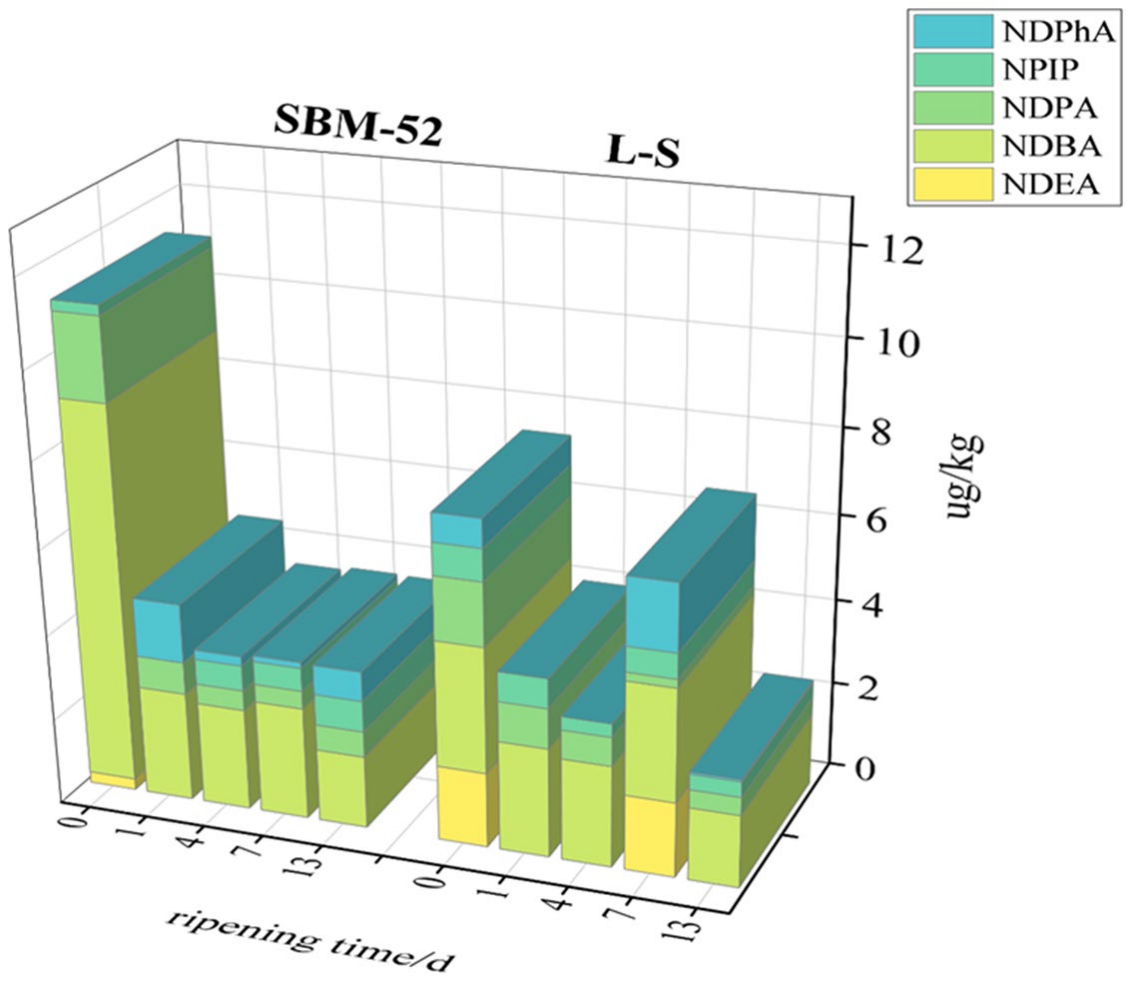
The impact of inoculating L-S strains and SBM-52 strains on the N-nitrosamines contents of cultured sausages throughout ripening.

N-nitrosamines (NAs) are easily created by secondary amines reacting with nitrifying agents such as nitrite, which might cause diabetes and cancer (Lu et al., 2022). NAs presence in fermented sausages in the maturation process was depicted in Figure 9. In our experiment, five nitrosamines were detected in fermented sausage, which were NDEA (N-nitrosodiethylamine), NDPA (N-nitrosodi-npropylamine), NPIP (N-nitrosopiperidine), NDBA (N-nitrosodibutylamine), and NDPhA (N-nitrosodiphenylamine). Studies have shown that there are various N-nitrosamines in fermented food, among which the most harmful are NDMA and NDEA (from group 2A) because they were classified as probable carcinogens, while NDPA and NDBA, and NPIP (from group 2B) are potential carcinogens and NDPhA from group 3 is unclassifiable as far as this category is concerned (Sun et al., 2017). Fortunately, NDMA was not detected in either fermented sausage. NDEA was detected only at 0 day of ripening in the SBM-52 sausages and 0 day and 7 days of ripening in the L-S sausages. NDBA is another primary N-nitrosamine found in fermented sausages, and its change trend was similar to NDPA. The concentrations of NDBA and NDPA in both sausages remained stable after a sharp decrease on the first day of ripening. The NPIP concentrations in the sausages cultured with L-S strains were 0.36 μg/kg lower than that in the SBM-52 sausages on day 13, the NPIP may be generated because of paprika and black pepper, containing pyrrolidine and piperidine (De Mey et al., 2017). The fluctuated trend in the concentrations of NDPhA in two sausages in maturation as illustrated in Figure 9, the NDPhA concentrations of the L-S group (0.05 μg/kg) were clearly lower than that of SBM-52 (0.69 μg/kg). As shown in Figure 9, the total concentrations of N-nitrosamine decreased first and then stabilized at about 3.70 μg/kg in the SBM-52 sausages, that varied from 2.59 μg/kg to 7.54 μg/kg in the L-S sausages. Finally, the total nitrosamine concentrations (3.40 μg/kg) of the L-S sausages were obviously lower than that (3.70 μg/kg) of the SBM-52 sausages. De Mey et al. (2014) suggested that the occurrence of NAs could be linked to the residual amounts of sodium nitrite, but there was no correlation was observed between the NAs and the BAs, so the decrease in NAs concentrations might be attributed to the reduced nitrite contents. In addition, Sun et al. (2023) also found that the SLPs of proteins located on the LAB cell wall could decrease the levels of NAs concentrations, so the NAs degradation may also be attributed to microbial function. Additionally, Ozbay (2022) found that the cooking process significantly affected the levels of all nitrosamine derivatives in different brands, ingredients, and types of salami. As a result, optimizing ripening process parameters to reduce the concentrations of NAs is also worth further research.

## 4. Conclusion

In this study, the fermented sausages were supplied with selected L-S strains to analyze the physicochemical characteristics of sausages in their ripening stage, and commercially available starter cultures SBM-52 as control. The results indicated that the inoculation of the mixed strain L-S rapidly reduced Aw and pH values in cultured sausages, helping to improve the lightness in sausages. The abilities of the L-S strains to deplete lipid oxidation were equivalent to the commercial SBM-52 strains. The NPN contents in L-S sausages were higher than that in SBM-52 sausages. The nitrite residues, BAs concentrations, and N-nitrosamine accumulations in the L-S-inoculated sausages were lower than in the SBM-52 sausages at the end of ripening. These findings imply that L-S strains would be suitable candidates for microbial culture starters in preparing cultured sausages, which would enhance the product quality.

## Data availability statement

The raw data supporting the conclusions of this article will be made available by the authors, without undue reservation.

## Author contributions

PH: methodology, investigation, data curation, and writing–original draft. UA: methodology and writing-review. TA: editing and investigation. LW: visualization and resources. JZ: conceptualization and funding acquisition. GN: editing and critical review. MS: software and investigation. YY: methodology, formal analysis, validation, and writing–review and editing. YZ: conceptualization, funding acquisition, supervision, and writing–review and editing. All authors contributed to the article and approved the submitted version.

## Funding

This research was funded by the key research and development project of Lyuliang high-level scientific and technological talents (Grant NO. 2022RC19), the teaching reform and innovation project of universities in Shanxi Province (Grant NO. J20221148), the Lyuliang key laboratory of agricultural product processing and functional food research (Grant NO. 2021ZDSY-1-47), the Natural Science Research Projects of Shanxi Province (20210302123400), and the Functional Food Technology System Construction Project in Shanxi Province (2022HX080).

## Conflict of interest

The authors declare that the research was conducted in the absence of any commercial or financial relationships that could be construed as a potential conflict of interest.

## Publisher’s note

All claims expressed in this article are solely those of the authors and do not necessarily represent those of their affiliated organizations, or those of the publisher, the editors and the reviewers. Any product that may be evaluated in this article, or claim that may be made by its manufacturer, is not guaranteed or endorsed by the publisher.
